# Microstructural Evolution and Mechanical Properties of AA2017 Aluminum Alloy Joints Produced by Rotary Friction Welding and TIG Welding

**DOI:** 10.3390/ma19163385

**Published:** 2026-08-09

**Authors:** Piotr Noga, Anna Kula, Marcel Wiewióra, Tomasz Skrzekut

**Affiliations:** Faculty of Non-Ferrous Metals, AGH University of Krakow, Mickiewicza 30 Av., 30-059 Krakow, Poland; kula@agh.edu.pl (A.K.); wiewiora@agh.edu.pl (M.W.); skrzekut@agh.edu.pl (T.S.)

**Keywords:** AA2017 aluminum alloy, rotational friction welding, TIG welding, microstructure evolution

## Abstract

**Highlights:**

- Rotary Friction Welding produced a fine-grained microstructure in AA2017 alloy.
- TIG welding produced a coarse-grained cast microstructure in the fusion zone as a result of solidification.
- RFW and TIG joints exhibited distinct hardness distributions and fracture mechanisms.
- RFW joints exhibit superior tensile strength and ductility compared to TIG joints.

**Abstract:**

This study investigates the influence of joining technology on the microstructural evolution and mechanical properties of joints produced from extruded AA2017 aluminum alloy rods. Rotary Friction Welding (RFW) was compared with conventional TIG welding to evaluate the effects of solid-state and fusion-based joining mechanisms. The joints were characterized using scanning electron microscopy (SEM), energy-dispersive spectroscopy (EDS), electron backscatter diffraction (EBSD), hardness measurements, tensile testing, and fracture analysis. TIG welding produced a coarse-grained cast microstructure within the fusion zone, whereas RFW generated a fine-grained microstructure formed through intense thermomechanical deformation accompanied by crystallographic texture evolution. These distinct microstructural characteristics resulted in markedly different mechanical behavior. The RFW joints achieved an ultimate tensile strength of 247 MPa and an elongation to failure of 12.5%, compared with 160 MPa and 1.7%, respectively, for the TIG-welded joints. The results demonstrate that the joint formation mechanism is the primary factor governing the microstructural evolution and mechanical performance of AA2017 alloy joints.

## 1. Introduction

The 2xxx series aluminum alloys are among the most widely used high-strength structural materials, with copper as their principal alloying element. Owing to their high strength-to-weight ratio, excellent fatigue resistance, and favorable combination of mechanical properties, these alloys are widely employed in the aerospace, automotive, and mechanical engineering industries, particularly for manufacturing components subjected to high service loads, such as shafts, axles, fasteners, and structural parts [1,2,3]. Among the 2xxx series alloys, AA2017 is one of the most widely used grades for highly loaded structural applications. It is typically supplied as extruded rods exhibiting a characteristic fibrous microstructure developed during hot extrusion, while its excellent mechanical properties are primarily attributed to thermomechanical processing [4,5,6,7].

Despite their attractive mechanical properties, 2xxx series aluminum alloys are generally regarded as difficult-to-weld materials. Their high copper content increases susceptibility to hot cracking and alloying element segregation during weld metal solidification, thereby making the production of sound welded joints particularly challenging. Furthermore, the high heat input associated with fusion welding promotes grain coarsening, microstructural evolution, and the transformation of strengthening phases, ultimately resulting in the deterioration of joint mechanical properties [8,9].

Gas Tungsten Arc Welding (GTAW/TIG) remains one of the most widely used joining techniques for aluminum alloys [10,11,12]. Owing to its excellent arc stability and precise control of weld bead geometry, this process has long been employed for the fabrication of critical aluminum structures. However, in Al–Cu alloys, complete melting followed by solidification leads to the formation of a coarse-grained cast microstructure, grain coarsening within the heat-affected zone (HAZ), and local heterogeneity in both chemical composition and the distribution of strengthening phases. Consequently, TIG-welded joints generally exhibit lower strength and ductility than the base material [13]. Another major limitation of TIG welding is the susceptibility of Al–Cu alloys to hot cracking. This phenomenon is associated with the presence of thin films of copper-rich liquid along grain boundaries during the final stage of solidification, combined with the action of solidification shrinkage stresses [14]. In addition, the high thermal conductivity of aluminum necessitates the application of relatively high heat input, resulting in a wider heat-affected zone and more extensive microstructural modification. These phenomena represent some of the principal limitations of conventional fusion welding techniques when applied to high-strength 2xxx series aluminum alloys [15].

Over the past two decades, solid-state joining technologies have advanced rapidly in response to the limitations of conventional fusion welding processes. Among these, Friction Stir Welding (FSW) has received considerable research attention owing to its ability to produce high-quality joints with superior mechanical properties by eliminating material melting during the joining process [16,17,18]. Numerous studies have demonstrated that, in 2xxx series aluminum alloys, FSW promotes significant grain refinement through dynamic recrystallization while suppressing the adverse phenomena commonly associated with fusion welding, including grain coarsening, alloying element segregation, and hot cracking [19,20,21,22,23,24]. Consequently, FSW has become one of the most extensively investigated and well-established solid-state joining techniques for high-strength aluminum alloys.

In contrast, considerably less attention has been paid to Rotary Friction Welding (RFW), despite its particular suitability for joining axisymmetric components such as rods, shafts, and tubes. As a solid-state joining process, RFW produces joints through the combined action of frictional heating and axial pressure without melting the material. As a result, defects commonly associated with fusion welding, including hot cracking, porosity, and alloying element segregation, are significantly reduced, while the extent of microstructural modification is minimized. Previous studies have demonstrated that RFW joints are characterized by the formation of a fine-grained dynamically recrystallized zone (DRZ), surrounded by a thermomechanically affected zone (TMAZ), which contributes to enhanced hardness and improved mechanical properties [25,26,27,28].

Although the advantages and limitations of RFW and fusion welding have been reported for several 2xxx aluminum alloys, a direct comparison between RFW and TIG welding for AA2017 alloy has not yet been comprehensively investigated. Therefore, the present study addresses this knowledge gap by providing a systematic comparison of the microstructural evolution, crystallographic texture, hardness distribution, tensile properties, and fracture behavior of AA2017 joints produced by Rotary Friction Welding and TIG welding.

## 2. Materials and Methods

**Figure 1:**
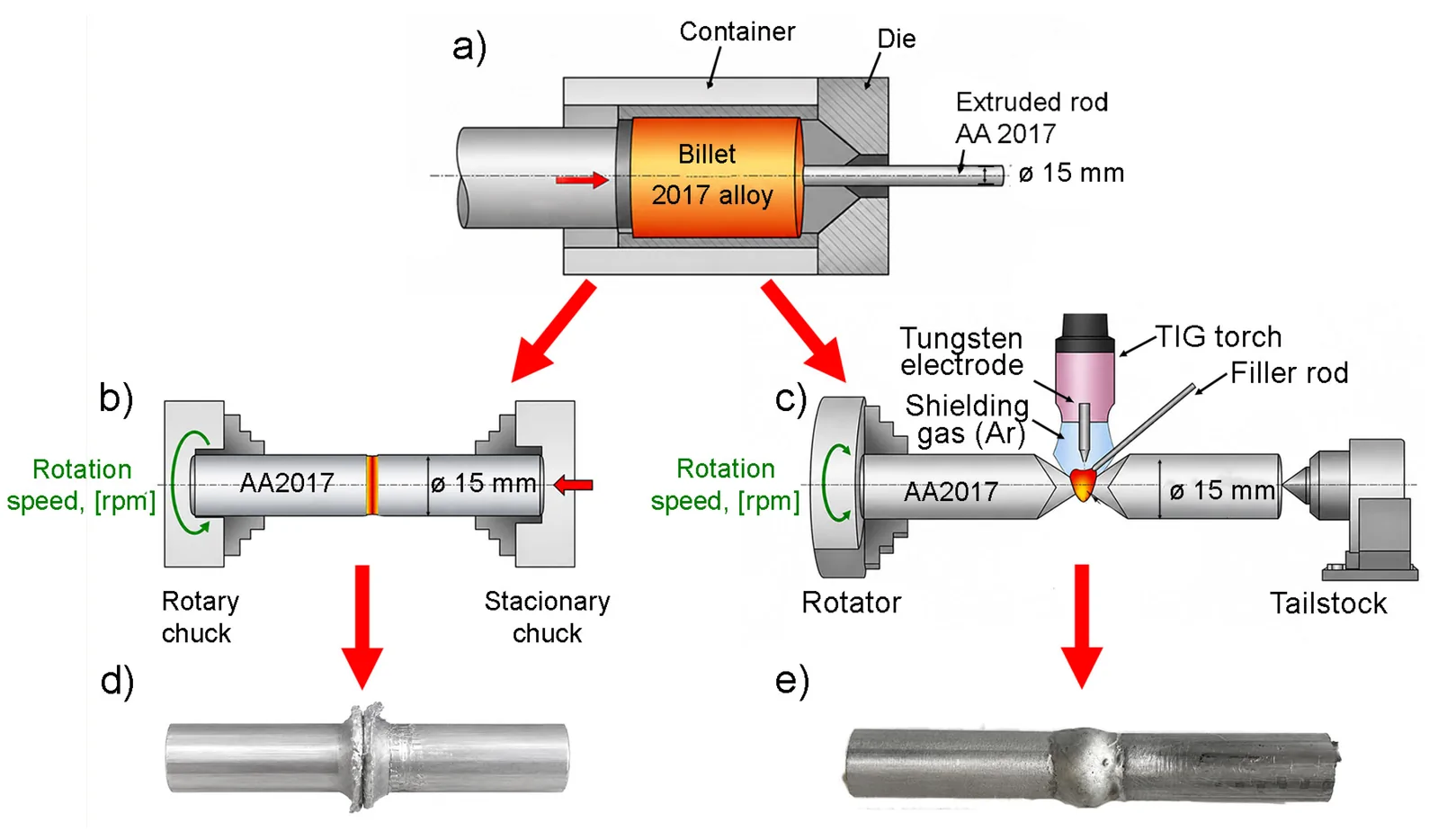
Schematic illustration of the experimental procedure: (**a**) hot extrusion of the AA2017 aluminum alloy; (**b**) Rotary Friction Welding (RFW); (**c**) TIG welding; (**d**) representative RFW joint; and (**e**) representative TIG-welded joint.

The experimental procedure adopted in this study is schematically illustrated in Figure 1. A commercial AA2017 aluminum alloy was used as the base material. Cylindrical billets with a diameter of 40 mm and a length of 80 mm were hot extruded at 350 °C to produce rods with a diameter of 15 mm (Figure 1a). No post-extrusion heat treatment was applied, and the extruded rods were used as the starting material for the production of joints by Rotary Friction Welding (RFW) and TIG welding. The chemical composition of the AA2017 alloy is listed in Table 1.

Rotary Friction Welding was performed on rods with a diameter of 15 mm (Figure 1b). During the process, one rod was rotated while the other remained stationary and was subjected to an axial force. Prior to welding, the rod end surfaces were machined and subsequently cleaned and degreased with acetone. The welding parameters were established on the basis of preliminary experiments using different combinations of friction and upsetting forces. Since only minor differences in the mechanical properties of the joints were observed, the parameter set providing the best mechanical performance was selected for further investigation. All RFW joints analyzed in the present study were produced using the same welding parameters. The friction force and upsetting force were 7 kN and 24.5 kN, respectively. The friction and upsetting times were 2.7 s and 1.9 s, respectively. The rotational speed was set to 900 rpm. The feed rate was 2.5 mm/s during the friction stage and 30 mm/s during upsetting. A representative RFW joint is shown in Figure 1d.

For comparison, joints were also produced by TIG welding. Welding was carried out on 15 mm diameter rods prepared with a conical butt joint geometry (Figure 1c). Both rod ends were machined to a conical shape, leaving a 1 mm root gap between the specimens. Prior to welding, the joint surfaces were cleaned and degreased with acetone. The process was performed using a Kemppi power source (Kemppi Oy, Lahti, Finland) under argon shielding gas with a flow rate of 15 L/min. A non-consumable tungsten electrode with a diameter of 2.4 mm and an AlMg4.5Mn filler wire with a diameter of 2.4 mm were used. The chemical composition of the filler wire is given in Table 1. Welding was performed using alternating current (AC) with a current of 100 A, a voltage of 18 V, and a frequency of 60 Hz. During welding, the specimens were mounted in a welding positioner and rotated at a constant speed corresponding to one revolution every 15 s, while the welding torch remained stationary. The total welding time was 50 s. All TIG joints analyzed in the present study were produced using the same welding parameters. A representative TIG-welded joint is presented in Figure 1e.

**Figure 2:**
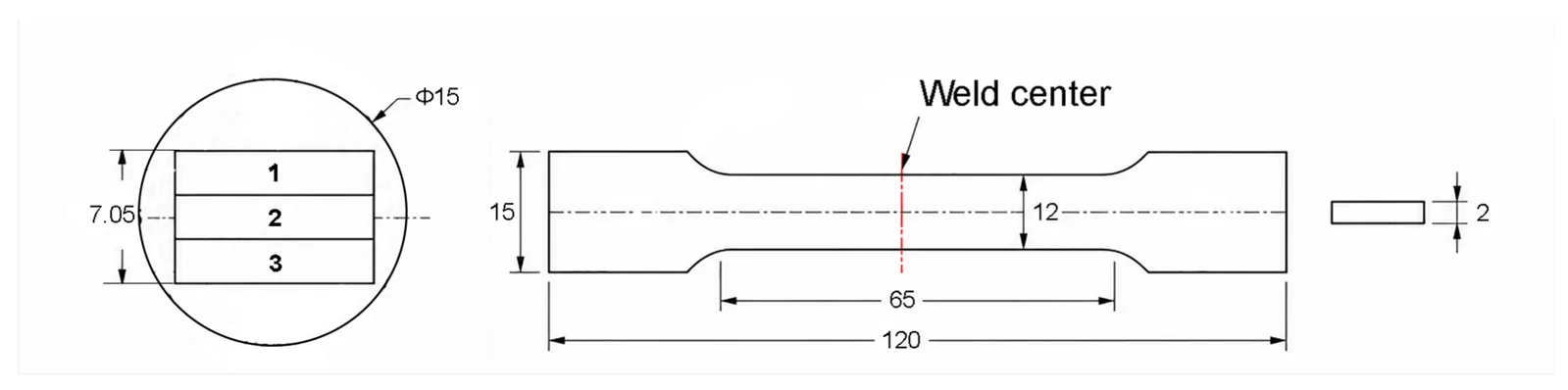
Geometry and dimensions of the tensile specimens together with a schematic illustration of their arrangement within the welded rod after the joining process.

The mechanical properties were evaluated by uniaxial tensile testing at room temperature using a Zwick/Roell Z050 (ZwickRoell GmbH & Co. KG, Ulm, Germany) universal testing machine. The tests were carried out at an initial strain rate of 8 × 10^−3^ s^−1^. Tensile specimens were prepared by electrical discharge machining (EDM) using a BP-05 wire-cut EDM machine and extracted parallel to the extrusion direction. For both the rotary friction welded and TIG-welded joints, the weld interface (RFW) and the fusion zone (TIG) were positioned at the center of the gauge length, ensuring that the entire joint region was located within the gauge section. The specimen geometry was selected to maximize the length of the joint within the gauge section. The specimens had a gauge length of 65 mm, a width of 12 mm, and a thickness of 2 mm, as shown in Figure 2. Strain was measured using an extensometer during the tensile tests, and the yield strength (Rp0.2) was determined using the 0.2% offset method. Three specimens were tested for each material condition, and the reported results represent the average values obtained from the measurements.

Microstructural characterization was carried out using a Hitachi SU-70 scanning electron microscope (SEM) (Hitachi High-Tech Corporation, Tokyo, Japan) equipped with Energy Dispersive Spectroscopy (EDS) and Electron Backscatter Diffraction (EBSD) detectors (Oxford Instruments, Abingdon, UK). In addition, the fracture surfaces of the tensile-tested specimens were examined by SEM.

Prior to SEM observations, the specimens were ground using SiC abrasive papers with progressively finer grit sizes up to 1200, followed by mechanical polishing with diamond suspensions of 9, 3, and 1 μm. Final polishing was performed using a 0.05 μm colloidal silica suspension. For EBSD analysis, the specimens were additionally subjected to final electrolytic polishing using A2 electrolyte (Struers, Ballerup, Denmark) at 15 V for 20 s to remove the mechanically deformed surface layer generated during specimen preparation and to obtain a surface suitable for diffraction pattern acquisition. EBSD maps were acquired using a field-emission scanning electron microscope equipped with an Oxford C-Swift+ EBSD detector. The step size was selected according to the local grain size and ranged from 0.1 to 2 μm. A step size of 0.1 μm was used for the fine-grained RFW zone, whereas larger step sizes, up to 2 μm, were used for the TIG regions containing substantially coarser grains. The original indexing rate ranged from 84% to 90% for the RFW maps and from 95% to 98% for the TIG maps. The EBSD data were processed using AZtecCrystal software (Oxford Instruments, Abingdon, UK). Solutions were extrapolated into non-indexed pixels only when at least five neighboring pixels provided a consistent solution. Following data cleaning, the fraction of indexed or reconstructed points reached approximately 95% for the RFW maps and 100% for the TIG maps. For the finest-grained RFW regions, a minimum grain-size criterion of 15 pixels per grain was applied. For maps acquired with a step size of 0.1 μm, this corresponds to a minimum equivalent grain diameter of approximately 0.44 μm. Two representative fields comprising more than 10,000 grains in total were included in the grain-size analysis. Hardness measurements were performed using the Vickers method under a load of HV2 with a dwell time of 10 s. Consecutive indentations were spaced at 1 mm intervals. Two-dimensional hardness maps were constructed from five parallel measurement lines distributed across the entire height of the joint. Additionally, a one-dimensional hardness profile was obtained from a single measurement line passing through the center of the joint, with the 0 mm position corresponding to the weld centerline (TIG) or the welding axis (RFW). Each reported hardness value corresponds to a single Vickers indentation.

## 3. Results

### 3.1. Microstructural Evolution of the Base Material and Welded Joints

**Figure 3:**
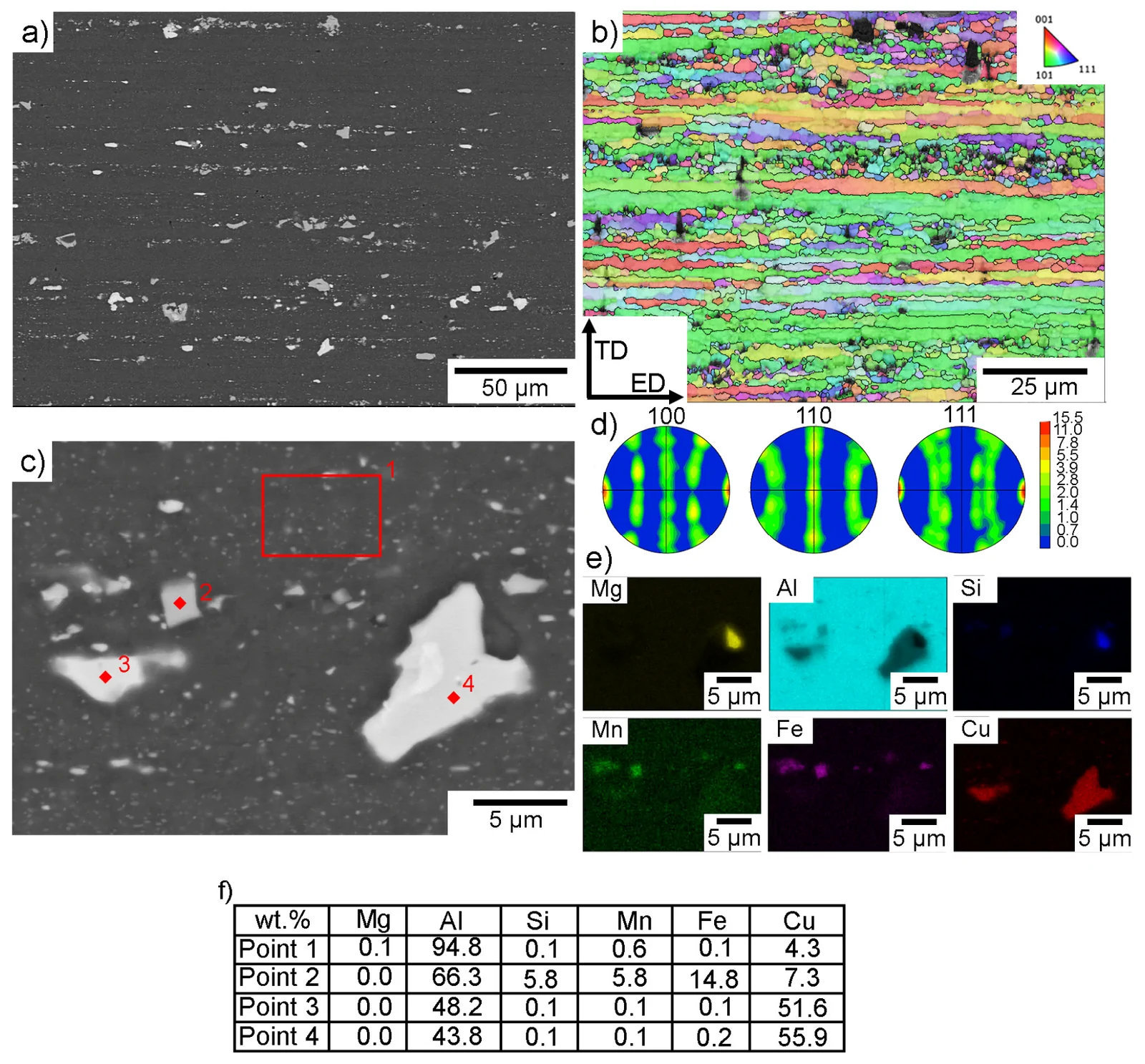
Microstructure of the extruded AA2017 aluminum alloy: (**a**,**c**) SEM micrographs (**b**) EBSD crystallographic orientation map with the extrusion direction (ED) indicated; (**d**) corresponding pole figures; (**e**) elemental distribution maps of Mg, Al, Si, Mn, Fe, and Cu; and (**f**) results of the point EDS chemical composition analysis performed at points indicated in (**c**).

The microstructure of the extruded AA2017 alloy served as the reference condition for evaluating the microstructural changes induced by the joining processes (Figure 3). Scanning electron microscopy observations revealed a homogeneous aluminum matrix containing numerous uniformly distributed intermetallic particles (Figure 3a). Most of these particles exhibited elongation parallel to the extrusion direction, indicating their deformation during thermomechanical processing. The characteristic fibrous microstructure was further confirmed by the crystallographic orientation map obtained by Electron Backscatter Diffraction (Figure 3b). The grains were distinctly elongated along the extrusion direction (ED), as shown in the EBSD orientation map (Figure 3b). This elongated grain morphology is characteristic of extruded aluminum alloys and reflects the material flow during the extrusion process. The presence of a preferred crystallographic orientation (texture) resulting from extrusion was further confirmed by the pole figures shown in Figure 3d.

To identify the observed intermetallic particles, point chemical composition analysis by Energy Dispersive Spectroscopy and elemental distribution mapping were performed (Figure 3c–f). The results revealed the presence of Cu-rich particles together with precipitates enriched in Fe, Mn, and Si. The point EDS analysis (Figure 3f) showed that points 3 and 4 correspond to Cu-rich particles, whereas point 2 is enriched in Fe, Mn, and Si. These intermetallic particles are characteristic of 2xxx series aluminum alloys and originate from the solidification process as well as subsequent thermomechanical processing.

**Figure 4:**
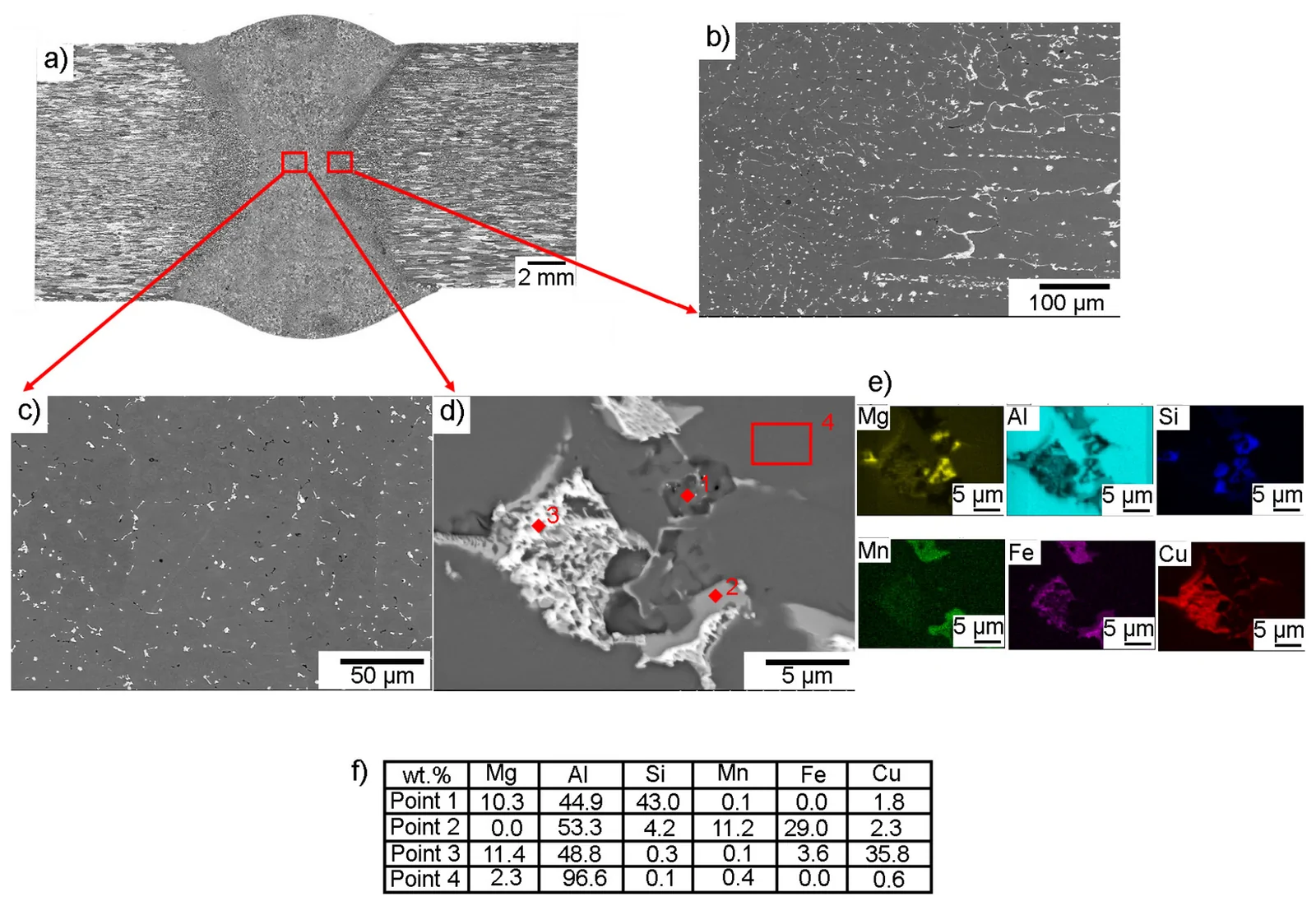
Microstructure of the TIG-welded joint: (**a**) macrostructure of the joint; (**b**,**c**) SEM micrographs; (**d**) SEM micrograph showing the locations selected for point EDS analysis and elemental mapping; (**e**) elemental distribution maps; and (**f**) results of the point EDS chemical composition analysis.

The TIG welding process resulted in a substantial modification of the joint microstructure due to local melting of the material followed by subsequent solidification (Figure 4). The macrostructure of the joint confirmed complete penetration and the formation of a sound weld bead (Figure 4a). No welding imperfections, such as cracks or incomplete penetration, were observed. SEM observations revealed a pronounced difference between the base material and the fusion zone (Figure 4b,c). While the base material retained the characteristic fibrous microstructure produced by hot extrusion, the fusion zone exhibited a typical cast microstructure formed during solidification of the molten metal.

Numerous intermetallic particles with different morphologies were observed within the fusion zone, predominantly in the interdendritic regions. EDS analysis and elemental distribution maps (Figure 4d–f) confirmed that the weld matrix consisted primarily of aluminum, whereas the intermetallic particles exhibited local enrichment in Cu, Mg, Mn, Si, and Fe. The heterogeneous distribution of alloying elements together with the presence of intermetallic particles of varying chemical composition suggests local compositional variations associated with the melting and solidification of the Al–Cu alloy.

**Figure 5:**
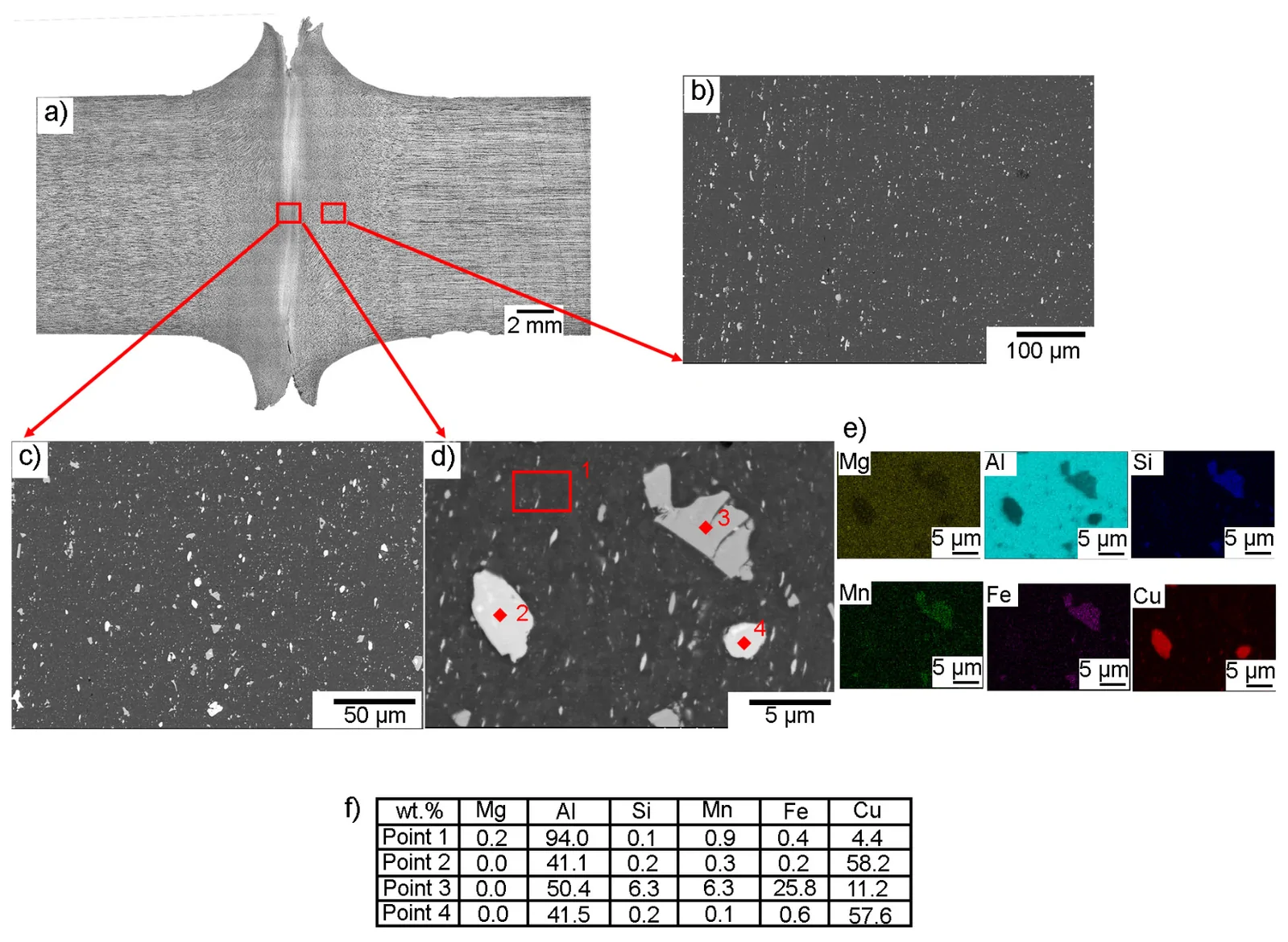
Microstructure of the Rotary Friction Welded (RFW) joint: (**a**) macrostructure of the joint; (**b**,**c**) SEM micrographs; (**d**) SEM micrograph showing the locations selected for point EDS analysis and elemental mapping; (**e**) elemental distribution maps; and (**f**) results of the point EDS chemical composition analysis.

A distinctly different microstructural evolution was observed in the joint produced by Rotary Friction Welding, where bonding is achieved in the solid state without material melting (Figure 5). The macrostructure confirmed the formation of a continuous joint across the entire cross-section of the rods (Figure 5a). Characteristic features of the joint include a well-defined weld interface and the flash formed as a result of intense plastic deformation during the welding process.

SEM observations revealed a microstructure characteristic of Rotary Friction Welding [28], in which the continuity of both the aluminum matrix and the intermetallic particles was preserved (Figure 5b,c). In contrast to the TIG-welded joint, no cast microstructure or microstructural features associated with melting and solidification were observed, confirming the solid-state nature of the joining process. EDS analysis and elemental distribution maps (Figure 5d–f) identified intermetallic particles enriched in Cu, Fe, Mn, and Si.

Compared with the base material, the RFW joint exhibited the disappearance of the characteristic banded distribution of intermetallic particles produced during extrusion and their more homogeneous distribution throughout the matrix. At the same time, the particles displayed a finer morphology while retaining a chemical composition comparable to that of the particles present in the base material. These findings indicate that Rotary Friction Welding did not result in any significant transformation of the intermetallic phases. Instead, the observed microstructural changes were primarily associated with severe plastic deformation and material flow during the welding process.

### 3.2. EBSD Analysis

**Figure 6:**
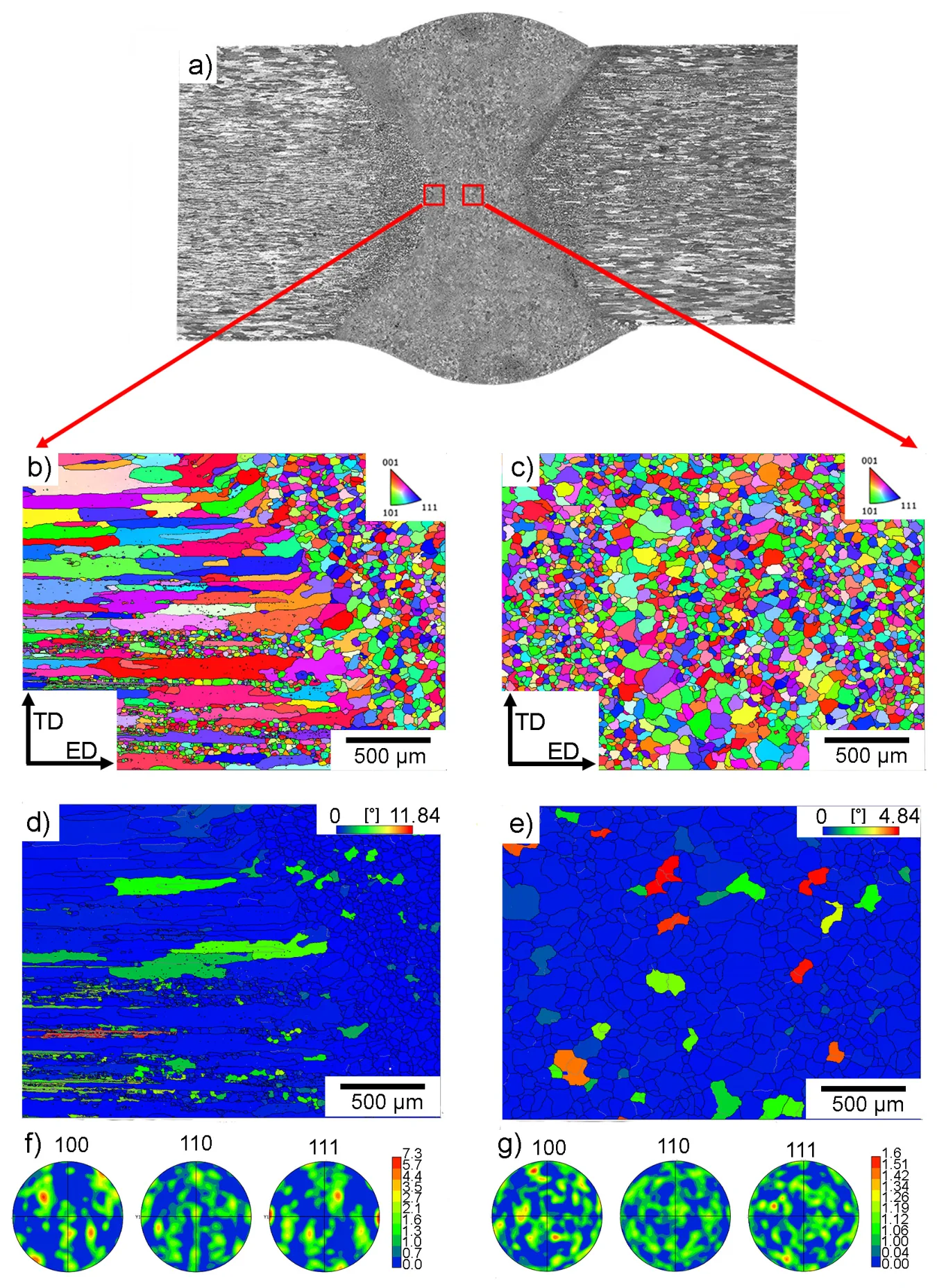
EBSD analysis of the TIG-welded joint: (**a**) macrostructure of the joint showing the regions selected for EBSD analysis; (**b**) inverse pole figure (IPF) orientation map across the fusion boundary; (**c**) IPF orientation map of the fusion zone; (**d**) grain orientation spread (GOS) map across the fusion boundary; (**e**) grain orientation spread (GOS) map of the fusion zone; and (**f**,**g**) corresponding pole figures.

The EBSD analysis of the TIG-welded joint provided a detailed insight into the microstructural evolution during welding and enabled the transition between the base material and the fusion zone to be clearly identified (Figure 6). The inverse pole figure (IPF) orientation map across the fusion boundary (Figure 6b) reveals a distinct transition from the fibrous microstructure inherited from the extrusion process to the equiaxed grain structure formed during solidification of the molten alloy. The base material retained its characteristic fibrous morphology, with elongated grains aligned parallel to the extrusion direction. As the fusion boundary was approached, this morphology gradually disappeared and was replaced by a more regular equiaxed grain structure. The average equivalent grain size in the vicinity of the fusion boundary was 25.0 ± 2.1 µm.

An even more pronounced microstructural transformation was observed in the central region of the fusion zone (Figure 6c). In contrast to the strongly oriented fibrous microstructure of the extruded material, this region consisted of equiaxed grains with a nearly random crystallographic orientation. Such a microstructure is typical of the fusion zone, where complete melting of the material followed by solidification results in the formation of equiaxed grains with a nearly random crystallographic orientation.

The average grain size within the fusion zone was approximately 40 μm, indicating substantial grain growth compared with the extruded material.

These results confirm that TIG welding leads to a complete transformation of the joint microstructure as a consequence of material melting and subsequent solidification. The differences between the individual joint regions are also reflected in the crystallographic texture. The pole figures presented in Figure 6d,e indicate that the crystallographic texture remains more pronounced in the vicinity of the fusion boundary than in the central region of the fusion zone. The reduced pole intensity observed in the center of the fusion zone indicates a weaker texture and a more random distribution of crystallographic orientations resulting from solidification. This indicates a more random distribution of crystallographic orientations in the grains formed during solidification. The GOS maps of both the fusion boundary and the fusion zone are dominated by low GOS values, indicating a low level of intragranular lattice distortion and a low density of stored dislocations. Only isolated grains exhibit locally increased GOS values.

**Figure 7:**
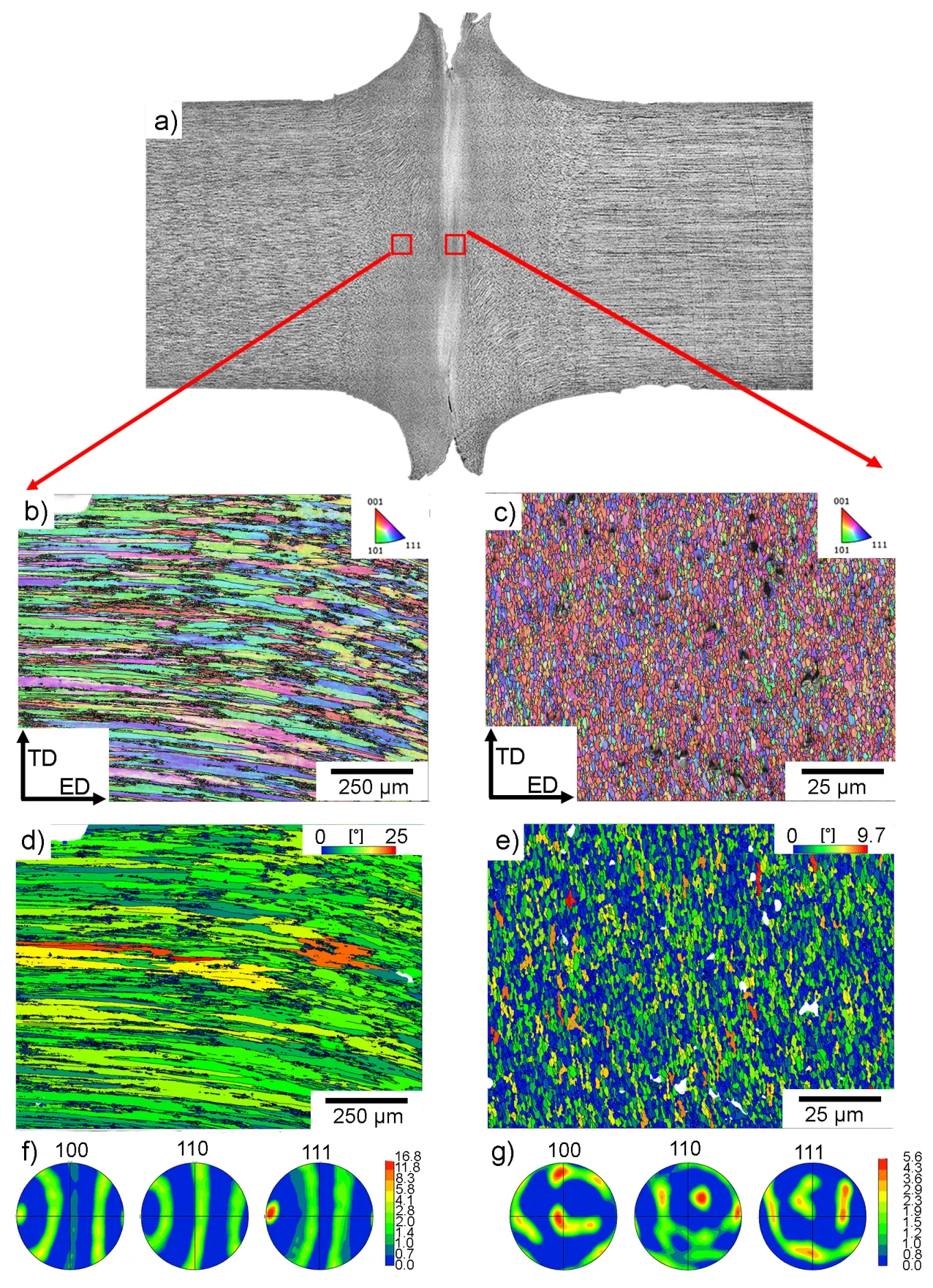
EBSD analysis of the Rotary Friction Welded (RFW) joint: (**a**) macrostructure of the joint showing the regions selected for EBSD analysis; (**b**) inverse pole figure (IPF) orientation map across the transition region; (**c**) IPF orientation map of the welding axis; (**d**) grain orientation spread (GOS) map across the transition region; (**e**) grain orientation spread (GOS) map of the welding axis; and (**f**,**g**) corresponding pole figures.

The EBSD analysis of the Rotary Friction Welded joint is presented in Figure 7. Owing to the fundamentally different microstructural evolution occurring in TIG- and RFW-produced joints, as well as the significant difference in grain size, the EBSD analyses were performed at different magnifications to provide a more detailed characterization of the microstructural changes within the individual joint regions.

The inverse pole figure orientation map of the transition region (Figure 7b) illustrates the gradual evolution of the microstructure from the base material towards the welding axis. In the unaffected base material, the characteristic fibrous microstructure with elongated grains aligned parallel to the extrusion direction was preserved. However, with decreasing distance to the welding axis, progressive grain refinement accompanied by a gradual transition from elongated to more equiaxed grains was observed. Simultaneously, the microstructure became increasingly aligned with the material flow induced by severe plastic deformation during the welding process.

The most significant microstructural changes were observed directly at the welding axis (Figure 7c). In this region, the original extrusion microstructure was replaced by a fine-grained equiaxed structure with an average grain size of approximately 1 μm. Such extensive grain refinement was associated with severe plastic deformation and elevated temperatures generated during the RFW process, creating conditions favorable for dynamic recrystallization. The GOS maps reveal clear differences between the thermomechanically affected region (Figure 7d) and weld zone (Figure 7c). The elongated grains adjacent to the weld center exhibit predominantly intermediate to high GOS values, indicating pronounced intragranular orientation gradients and a relatively high density of stored dislocations resulting from severe plastic deformation. In contrast, the fine equiaxed grains in the weld center are characterized mainly by low GOS values with only isolated grains exhibiting elevated GOS. This indicates a substantially lower level of intragranular lattice distortion and suggests that the dynamic recovery and recrystallization occurring during Rotary Friction Welding effectively reduced the stored deformation energy.

Compared with the TIG-welded joint, where the average grain size within the fusion zone was approximately 40 μm, the RFW joint exhibited a substantially finer microstructure. Furthermore, a distinct change in the preferred grain orientation relative to the base material was observed at the welding axis, indicating crystallographic reorientation caused by intense material flow during welding. These observations are further supported by the pole figures presented in Figure 7d,e. While the transition region partially retained the texture inherited from the extrusion process, the welding axis exhibited a pronounced change in the crystallographic orientation distribution, confirming the microstructural evolution occurring during Rotary Friction Welding.

### 3.3. Tensile Properties

**Figure 8:**
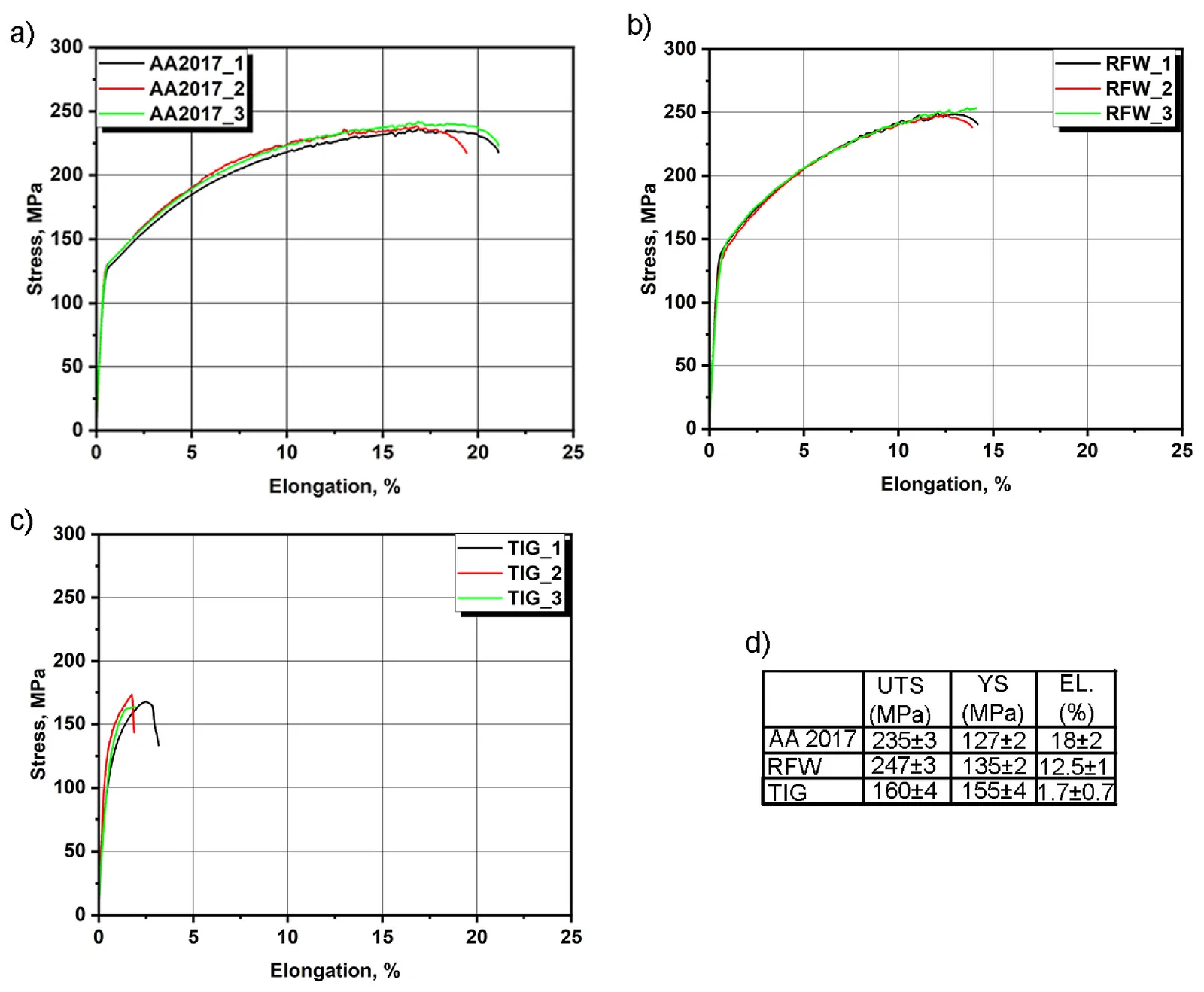
Tensile test results: (**a**) stress–strain curves of the extruded AA2017 alloy; (**b**) stress–strain curves of the RFW joints; (**c**) stress–strain curves of the TIG-welded joints; and (**d**) comparison of the average ultimate tensile strength (UTS), yield strength (YS), and elongation to failure (EL).

The microstructural changes observed in the investigated joints were directly reflected in their mechanical properties. Figure 8 presents the results of the tensile tests performed on the extruded AA2017 alloy and on the joints produced by RFW and TIG welding. All stress–strain curves exhibited good repeatability, confirming the stability of both the applied processing parameters and the resulting joints.

The extruded material exhibited an average ultimate tensile strength (UTS) of 235 MPa, a yield strength (YS) of 127 MPa, and an elongation to failure (EL) of 18% (Figure 7a,d). The most favorable combination of strength and ductility was obtained for the RFW joints. Their average UTS reached 247 MPa, while the YS increased to 135 MPa. Although the elongation to failure decreased to 12.5%, the material retained a high capacity for plastic deformation. These results indicate that Rotary Friction Welding not only preserved the mechanical properties of the extruded material but also resulted in a slight improvement in tensile strength.

A markedly different mechanical response was observed for the TIG-welded joints. In this case, the average UTS decreased to 160 MPa, whereas the elongation to failure was reduced to only 1.7%. At the same time, the YS reached 155 MPa, representing the highest value among all investigated material conditions. This combination of properties indicates a substantial reduction in the material’s ability to accommodate further plastic deformation after yielding, which is a direct consequence of the microstructural changes associated with melting and solidification during the TIG welding process. The fracture locations observed during tensile testing were consistent with the measured mechanical properties. All RFW specimens fractured outside the weld center, indicating that the weld region was not the weakest part of the joint. In contrast, all TIG-welded specimens fractured along the fusion boundary, which represented the preferential crack initiation site.

### 3.4. Fractographic Analysis

**Figure 9:**
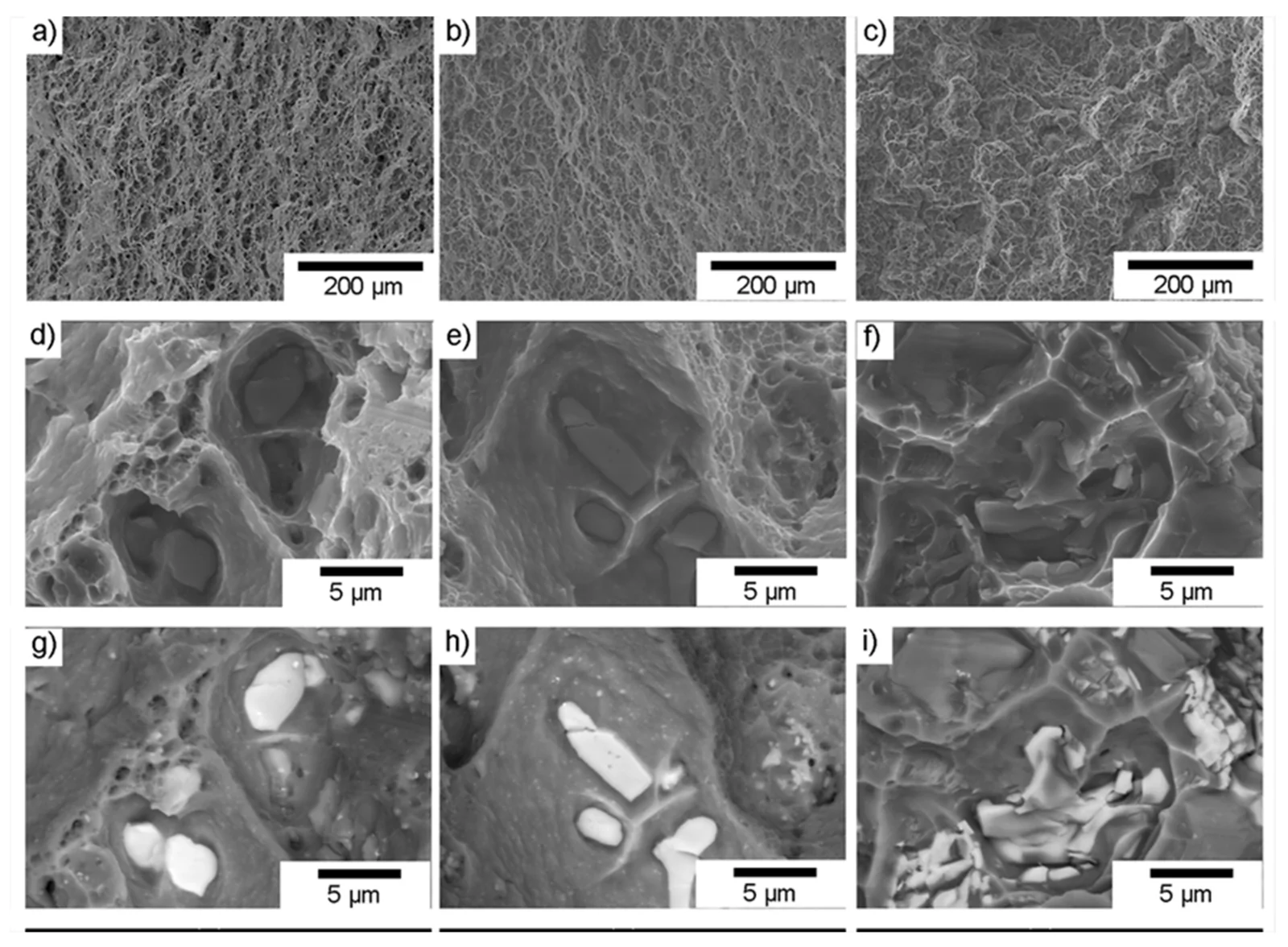
Fracture surfaces after tensile testing: (**a**–**c**) secondary electron (SE) micrographs; (**d**–**f**) higher-magnification SE micrographs; and (**g**–**i**) backscattered electron (BSE) micrographs. (**a**,**d**,**g**) extruded AA2017 alloy; (**b**,**e**,**h**) RFW joint; and (**c**,**f**,**i**) TIG-welded joint.

The microstructural changes occurring in the investigated joints were also reflected in their fracture behavior during tensile testing. Figure 9 presents SEM images of fracture surfaces of the extruded AA2017 alloy and of the joints produced by RFW and TIG welding. The fracture surface of the base material (Figure 9a,d) exhibited the characteristic features of ductile fracture, with numerous deep dimples indicating extensive plastic deformation prior to crack initiation and propagation [29].

BSE observations (Figure 9g) revealed the presence of intermetallic particles both within the dimples and on the fracture surface. These particles acted as local stress concentrators, promoting microvoid nucleation followed by their growth and coalescence during fracture. This fracture mechanism is characteristic of aluminum alloys containing a high-volume fraction of intermetallic particles and is consistent with the microstructural observations of the extruded material.

A similar fracture morphology was observed for the RFW joint (Figure 9b,e). The fracture surface also exhibited numerous ductile dimples; however, compared with the base material, they were generally finer and more uniformly distributed. The BSE micrographs (Figure 9h) revealed intermetallic particles embedded within the aluminum matrix, although their presence did not alter the dominant ductile fracture mechanism. These observations indicate that, despite the extensive microstructural refinement induced by Rotary Friction Welding, the material retained a high capacity for plastic deformation prior to fracture.

A distinctly different fracture morphology was observed for the TIG-welded joint (Figure 9c,f). Compared with both the base material and the RFW joint, the fracture surface contained considerably fewer dimples and was dominated by extensive flat regions and cleavage-like facets, indicating a more brittle fracture mode. The corresponding BSE micrographs (Figure 9i) confirmed the presence of intermetallic particles; however, the surrounding aluminum matrix exhibited a substantially lower degree of plastic deformation.

The distinctly different fracture morphology directly reflects the microstructural changes introduced by the TIG welding process, particularly the formation of a cast microstructure and the altered distribution of intermetallic particles. The reduced number of dimples together with the predominance of flat fracture regions indicates a significantly diminished capacity for plastic deformation, which is fully consistent with the tensile test results, where the TIG-welded joints exhibited the lowest elongation to failure.

### 3.5. Hardness Distribution

**Figure 10:**
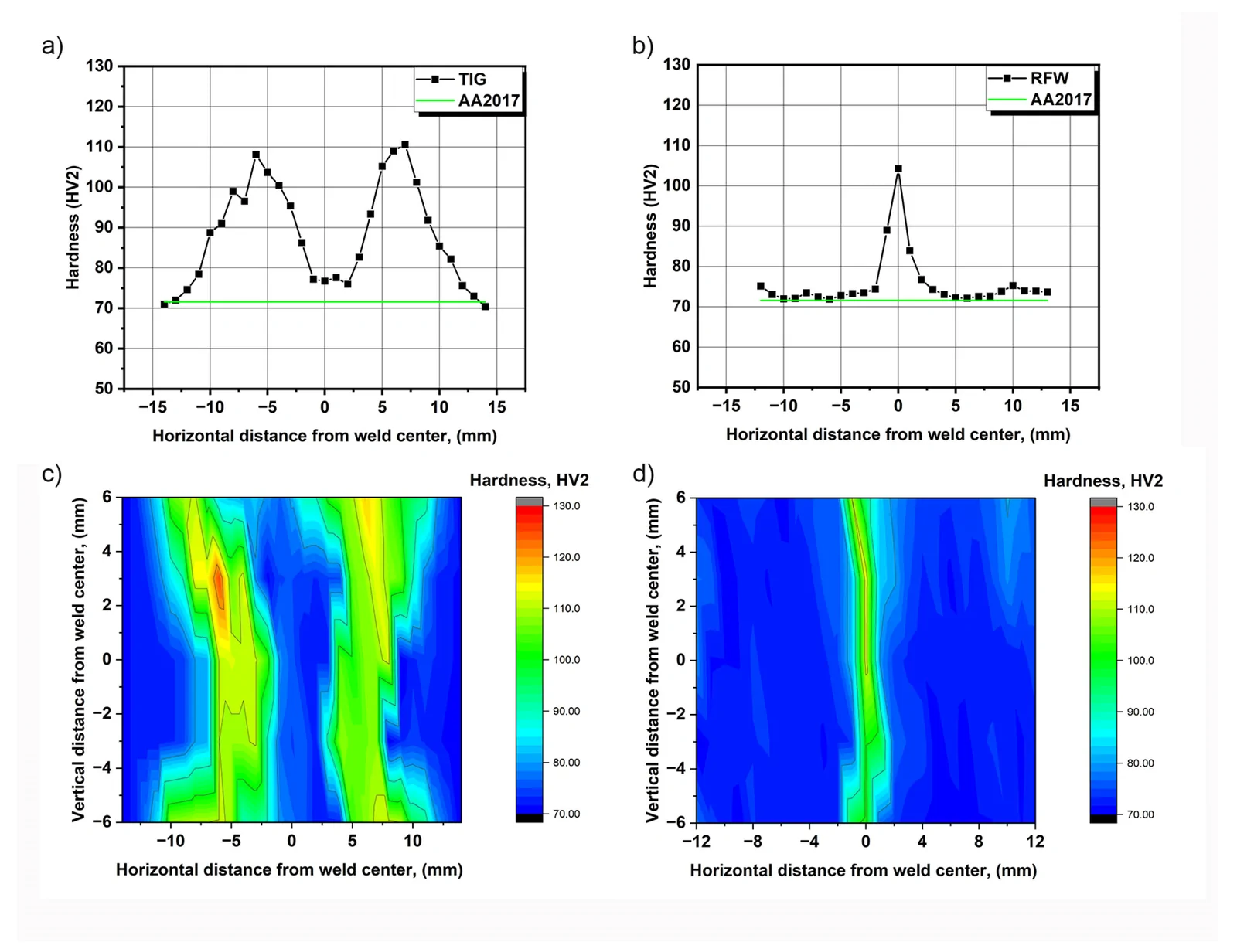
Vickers hardness (HV2) distribution: (**a**) hardness profile measured along the centerline of the TIG-welded joint; (**b**) hardness profile measured along the centerline of the RFW joint; (**c**) two-dimensional hardness map of the TIG-welded joint; and (**d**) two-dimensional hardness map of the RFW joint.

The effect of the applied joining techniques on the material properties was further evaluated by analyzing the hardness distribution across the transverse cross-sections of the joints. Figure 10 presents the hardness profiles and two-dimensional hardness maps for the TIG- and RFW-produced joints. For comparison, the average hardness of the extruded AA2017 base material, approximately 71HV2, is also indicated.

The hardness profiles shown in Figure 10a,b were measured along the centerline of the joints and were selected as representative of the hardness distribution across the weld. The corresponding two-dimensional hardness maps shown in Figure 9c,d were constructed using measurements performed along five parallel measurement lines distributed across the entire joint cross-section.

The TIG-welded joint (Figure 10a,c) exhibited a distinctly heterogeneous hardness distribution associated with the different microstructural regions formed during welding. The highest hardness values, exceeding 110 HV2, were recorded on both sides of the weld center, whereas the hardness within the central region of the fusion zone decreased to values comparable to those of the base material. The two-dimensional hardness map confirms the presence of two symmetrical bands of increased hardness located on either side of the weld center, reflecting the complex microstructural changes occurring within the heat-affected zone and the adjacent transition regions.

A markedly different hardness distribution was observed for the RFW joint (Figure 10b,d). In this case, the maximum hardness, approximately 105HV2, was measured directly at the welding axis. With increasing distance from the joint center, the hardness rapidly decreased to values comparable to those of the base material. The corresponding two-dimensional hardness map indicates that the region of increased hardness is confined to a narrow central zone, corresponding to the area of severe plastic deformation and microstructural refinement.

A comparison of the two joining techniques demonstrates that the hardness distribution is directly governed by the joint formation mechanism. In TIG welding, local melting followed by solidification produces a relatively wide region with heterogeneous mechanical properties. In contrast, the microstructural changes induced by Rotary Friction Welding are confined primarily to the narrow region surrounding the welding axis, where severe plastic deformation results in significant grain refinement and a local increase in hardness.

## 4. Discussion

The findings of the present study clearly demonstrate that the joint formation mechanism is the primary factor governing the final microstructure and mechanical performance of AA2017 alloy joints. The two joining techniques investigated resulted in fundamentally different microstructural evolution pathways. In TIG welding, joint formation involved complete melting of both the base material and the filler metal, followed by solidification of the molten pool. In contrast, Rotary Friction Welding is a solid-state joining process in which bonding is achieved through the combined effects of severe plastic deformation and localized frictional heating.

The distinctly different thermal and deformation conditions associated with these two joining processes were directly reflected in both the microstructural evolution and the resulting mechanical behavior of the joints. Whereas TIG welding produced a cast-like microstructure characteristic of solidification processes, the RFW joint preserved the metallurgical continuity of the material while undergoing extensive microstructural refinement driven by plastic deformation. The significance of solid-state processing has also been emphasized by Won et al. [30], who identified the absence of material melting as one of the principal advantages of friction welding technologies, as it eliminates phenomena commonly associated with conventional fusion welding, including elemental segregation, solidification cracking, and the formation of coarse-grained cast microstructures.

The extruded material served as the reference condition for evaluating the microstructural changes induced by the joining processes. It exhibited the characteristic fibrous microstructure typical of heavily plastically deformed aluminum alloys, with elongated grains aligned parallel to the extrusion direction. EBSD analysis confirmed the presence of a pronounced crystallographic texture inherited from the extrusion process, which is characteristic of this class of aluminum alloys [31]. The preserved grain orientation and directional morphology reflected the deformation history of the material and provided a reliable reference for assessing the extent of microstructural transformation induced by the joining processes.

A fundamentally different microstructural evolution was observed in the TIG-welded joint. Within the heat-affected zone (HAZ), the elongated grain morphology inherited from the extrusion process was preserved. However, exposure to the welding thermal cycle resulted in grain coarsening, with the average grain size increasing from approximately 8 µm in the base material to about 25 µm. This coarsening resulted from exposure to the elevated temperatures of the welding thermal cycle, which promoted grain boundary migration.

Even more pronounced microstructural changes were observed within the fusion zone, where complete melting and subsequent solidification resulted in the formation of a new cast microstructure. The resulting fusion zone was characterized by an equiaxed grain structure with an average grain size of approximately 40 µm ± 3 µm.

Comparable observations were reported by Milčić et al. [32] in their comparative study of FSW, MIG, and TIG welding of AA2024-T351 alloy. Their study demonstrated that the welding process eliminates the elongated microstructure characteristic of plastically worked material and gives rise to a cast microstructure composed of coarser grains with a modified distribution of intermetallic particles. They further showed that the extent of these microstructural changes is governed primarily by the welding thermal cycle and the cooling rate during weld solidification, which is fully consistent with the findings of the present study.

A fundamentally different microstructural evolution was observed in the RFW joint. At the welding axis, the average grain size decreased to approximately 1 μm, representing nearly an eightfold refinement relative to the extruded material and an approximately fortyfold reduction compared with the TIG fusion zone. Such extensive grain refinement indicates that dynamic recrystallization, promoted by the combined effects of elevated temperature and severe plastic deformation, was the dominant mechanism responsible for microstructural reconstruction.

A similar mechanism has been reported for friction-welded AA2024 and AA2014 alloys [33,34], in which a dynamically recrystallized zone (DRZ) composed of ultrafine equiaxed grains was identified and surrounded by a thermomechanically affected zone. As the distance from the welding axis increased, grain size gradually increased owing to the progressive reduction in plastic deformation and the diminishing influence of the thermal cycle.

The same trend was identified in the AA2017 joints investigated in the present study. While the transition region partially retained the extrusion-induced microstructure, the welding axis was completely transformed into a fine-grained structure produced by dynamic recrystallization. These observations demonstrate that RFW involves not merely local material displacement but rather extensive microstructural reconstruction within the region subjected to the highest thermomechanical loading. It should be noted that direct comparison of grain size values reported for different 2xxx aluminum alloys should be made with caution, as differences in chemical composition may influence the dynamic recrystallization behavior and the resulting microstructural evolution. Therefore, the literature data cited above are intended only to provide a qualitative comparison of the general microstructural evolution associated with Rotary Friction Welding.

Further insight into the mechanisms governing microstructural evolution during RFW was provided by the crystallographic orientation analysis. The IPF maps revealed a gradual evolution of grain orientation from the base material towards the welding axis. Although remnants of the extrusion texture were still preserved within the transition region, the welding axis exhibited a pronounced reorganization of crystallographic orientations associated with the intense flow of plastically deformed material.

The observed grain reorientation was a direct consequence of the deformation conditions prevailing during Rotary Friction Welding. At the welding axis, the material experienced intense shear deformation under elevated temperatures, resulting in the formation of a fine-grained microstructure whose crystallographic orientation was governed by the direction of material flow. A similar mechanism was described in Ref. [35], where the crystallographic orientation of grains within the dynamically recrystallized zone was shown to reflect the direction of material flow and deformation during the friction welding process.

The present findings indicate that RFW affects not only grain refinement but also the crystallographic orientation of the grains and the overall texture. The observed texture evolution is associated with the material flow and deformation conditions prevailing during the welding process.

The distinctly different microstructural evolution observed in the two joining processes was directly reflected in the hardness distributions of the corresponding joints. In the RFW joint, the highest hardness values, reaching approximately 105 HV2, were recorded at the welding axis, whereas the average hardness of the base material was approximately 70 HV2. Notably, the hardness maximum coincided with the region exhibiting the greatest degree of grain refinement, where the average grain size was approximately 1 μm. This correlation suggests that the increased hardness is consistent with grain boundary strengthening, as described by the Hall–Petch relationship [36]. However, the Hall–Petch relationship is referred to here only as a qualitative explanation of the observed behavior, and no quantitative Hall–Petch analysis was performed in the present study. Grain refinement increases the density of grain boundaries, which impede dislocation motion and consequently enhance the material’s resistance to plastic deformation.

The present results are consistent with previous studies showing that severe plastic deformation promotes grain refinement and dislocation accumulation, leading to enhanced hardness and mechanical properties. Similar strengthening mechanisms have also been reported in the literature [37]. A similar relationship has been reported for RFW joints of AA2024 alloy [35], the high hardness in DRZ is mainly caused by fine grain strengthening and the precipitation of GPB zones. The authors demonstrated that this region was characterized not only by the smallest grain size but also by the highest degree of microstructural reconstruction resulting from severe thermomechanical loading. Likewise, Bauri et al. [38] reported that the hardness increase in friction-welded 2xxx series aluminum alloys is primarily associated with the formation of a fine-grained microstructure produced by dynamic recrystallization.

The present results are in good agreement with these observations. Grain refinement was identified as the dominant factor responsible for the hardness increase in the RFW joint, as the hardness maximum corresponded closely to the region containing the finest grains identified by EBSD analysis. Both the hardness profile and the two-dimensional hardness map further demonstrated that the hardened region was confined almost exclusively to the welding axis. At only a short distance from this region, the hardness gradually returned to values characteristic of the base material.

The highly localized hardness distribution indicates that the hardness increase in the RFW joint is associated with the region subjected to the highest plastic deformation. A similar trend was reported in Ref. [34], where the width of the hardened region closely corresponded to the extent of the dynamically recrystallized zone and was shown to depend on the welding parameters.

A fundamentally different hardness distribution was obtained for the TIG-welded joints. In this case, the highest hardness values, approximately 110 HV2, were not located within the center of the fusion zone but on either side of the fusion boundary. Interestingly, EBSD analysis revealed that this region was characterized by an average grain size of approximately 25 μm, whereas the average grain size within the center of the fusion zone reached approximately 40 μm.

These findings indicate that the increased hardness observed near the fusion boundary was not associated with grain refinement, as in the RFW joint, but rather resulted from different microstructural mechanisms operating during the welding thermal cycle. Additional insight was provided by the EDS analysis, which revealed the presence of particles enriched in Cu, Mg, Mn, Fe, and Si both within the fusion zone and in its immediate vicinity.

The presence of copper within the fusion zone confirms extensive mixing between the AA2017 base material and the AlMg4.5Mn filler wire during welding. It is therefore reasonable to assume that exposure to the elevated welding temperatures promoted partial dissolution of the original intermetallic particles, followed by their re-precipitation during cooling. Consequently, the local hardness maximum observed near the fusion boundary should be attributed primarily to changes in the intermetallic particle population and the local supersaturation of the aluminum matrix, rather than to grain size variation. Similar phenomena were reported by Milčić et al. [32] in their comparative study of AA2024 alloy welded using FSW, MIG, and TIG processes. They demonstrated that the mechanical properties of the individual joint regions result from the combined effects of grain growth, redistribution of alloying elements, and transformations within the intermetallic particle system occurring during melting and solidification. The most pronounced property variations were observed in the vicinity of the fusion boundary, where the material experiences the most complex thermal cycle. The findings of the present study are in excellent agreement with these observations. The hardness maximum in the TIG joint was likewise located near the fusion boundary, whereas the center of the fusion zone exhibited hardness values slightly exceeding those of the base material. This confirms that, in fusion welding processes, the distribution of mechanical properties is governed primarily by local phase transformations and the thermal history of the individual regions of the joint.

The AlMg4.5Mn filler wire was selected because of its excellent weldability, widespread availability, and favorable mechanical properties. It should be noted, however, that the use of an Al–Mg–Mn filler metal with an Al–Cu base alloy modifies the local chemical composition of the fusion zone and may affect its microstructure and mechanical properties. Therefore, the comparison between the TIG and RFW joints reflects both the influence of the different joining mechanisms and the influence of compositional differences, which should be regarded as a limitation of the present study.

The differences in microstructural evolution and hardness distribution were directly reflected in the tensile properties. Among all the investigated material conditions, the RFW joints exhibited the most favorable mechanical performance, achieving an ultimate tensile strength of 247 MPa and an elongation to failure of 12.5%. These results demonstrate that Rotary Friction Welding preserves a favorable balance between strength and ductility. This behavior is a direct consequence of the joint formation mechanism characteristic of RFW. The absence of material melting preserves the metallurgical continuity of the aluminum matrix while minimizing the detrimental effects associated with solidification. At the same time, severe plastic deformation promotes dynamic recrystallization, producing a fine-grained microstructure that enhances the material’s resistance to deformation through grain boundary strengthening. As a result, the RFW joint exhibited higher tensile strength than the extruded material while retaining a high level of ductility.

In contrast, the TIG-welded joints exhibited an ultimate tensile strength of approximately 160 MPa and an elongation to failure of only 1.7%. This pronounced reduction in ductility was primarily associated with the extensive microstructural reconstruction induced by melting and subsequent solidification. The formation of a coarse-grained cast microstructure, together with changes in the distribution of intermetallic particles and local variations in chemical composition, significantly reduced the material’s ability to accommodate plastic deformation prior to crack initiation. It is noteworthy that, despite exhibiting the highest yield strength, approximately 155 MPa, the TIG-welded joints displayed only a very limited capacity for further plastic deformation once yielding had occurred. This indicates that an increased resistance to the onset of plastic deformation did not translate into improved overall structural performance of the joint. For components subjected to dynamic loading or requiring efficient energy absorption, the microstructural evolution associated with the RFW process proved to be considerably more advantageous.

Further evidence of the different mechanical responses of the two joint types was provided by the fracture surface analysis. Both the extruded material and the RFW joints exhibited the ductile fracture morphology characteristic of 2xxx series aluminum alloys, in which failure proceeds through the nucleation, growth, and coalescence of microvoids. In the base material, the fracture surface was characterized by numerous deep dimples, indicating extensive plastic deformation prior to final fracture. A similar fracture mechanism was observed in the RFW joint; however, the dimples were generally finer and more uniformly distributed. This fracture morphology is consistent with the finer and more homogeneous microstructure produced by dynamic recrystallization. The presence of intermetallic particles within the dimples, as revealed by the BSE micrographs, further confirmed that microvoid nucleation occurred predominantly at hard second-phase particles.

This fracture behavior is characteristic of the ductile failure mechanism commonly observed in 2xxx series aluminum alloys, involving the nucleation of microvoids through particle fracture or interfacial decohesion between intermetallic particles and the aluminum matrix, followed by void growth and eventual coalescence into the final fracture surface. A similar mechanism was reported for AA2024-T351 alloy [39], where intermetallic particles were identified as the primary sites for fracture initiation under tensile loading.

The present results indicate that the same fracture mechanism operated in the RFW joints of AA2017 alloy, while the preservation of a ductile fracture morphology confirms the high ductility retained by this type of joint. In contrast, the TIG-welded joints exhibited a distinctly different fracture morphology, characterized by a markedly lower density of dimples together with a greater proportion of flat regions and cleavage-like facets. Such features indicate earlier crack initiation and a reduced ability of the material to dissipate energy through plastic deformation.

The fracture behavior of the TIG joints is fully consistent with the tensile test results, in which these joints exhibited both the lowest ultimate tensile strength and the smallest elongation to failure among all the investigated material conditions. These findings demonstrate that the detrimental microstructural changes associated with the TIG welding process influence not only the local distribution of mechanical properties but also the mechanisms governing crack initiation and propagation.

The comprehensive experimental program, combining SEM observations, EDS chemical analysis, EBSD characterization, hardness mapping, tensile testing, and fractographic analysis, enabled the relationship between the joining technology and the resulting microstructure and mechanical properties to be established unequivocally.

As a solid-state joining process, RFW produced a fine-grained microstructure through dynamic recrystallization. The resulting grain refinement, local modification of the crystallographic texture, and preservation of matrix continuity enabled the production of joints exhibiting high strength while retaining substantial ductility. The hardness increase observed at the welding axis was directly associated with the dynamically recrystallized zone and is consistent with grain boundary strengthening described by the Hall–Petch relationship.

In contrast, TIG welding, owing to the presence of a liquid phase, resulted in complete microstructural reconstruction and the formation of regions with markedly different characteristics. Solidification produced a coarse-grained cast microstructure accompanied by significant changes in both the distribution and composition of intermetallic particles. Consequently, the TIG joints exhibited reduced tensile strength, severely limited ductility, and a more brittle fracture response.

Overall, the present findings demonstrate that, for 2xxx series aluminum alloys such as AA2017, the joint formation mechanism plays a decisive role in determining the final properties of the material after joining. Rotary Friction Welding therefore represents an attractive alternative to conventional fusion welding techniques, as it enables the production of joints with a more favorable combination of mechanical properties while minimizing the detrimental microstructural changes associated with processes involving a liquid phase.

## 5. Conclusions

1. Rotary Friction Welding (RFW) promotes a fundamentally different and mechanically more favorable microstructural evolution in AA2017 alloy than TIG welding. At the welding axis, RFW produced a fine-grained microstructure characteristic of an intensely thermomechanically transformed region, with an average grain size of approximately 1 μm. In contrast, TIG welding generated a coarse-grained cast microstructure with an average grain size of approximately 40 μm as a consequence of material melting and subsequent solidification.
2. EBSD analysis demonstrated that RFW not only produced extensive grain refinement but also induced crystallographic texture evolution associated with the direction of material flow during welding. The changes in grain morphology and crystallographic orientation at the welding axis indicate the occurrence of intense plastic deformation and dynamic recrystallization. At the same time, EDS analysis confirmed that the intermetallic particles retained the chemical characteristics of those present in the base material, with no evidence of significant compositional modification during the welding process.
3. The hardness distribution was directly governed by the microstructural evolution associated with each joining process. In the RFW joint, the maximum hardness (~105 HV2) was recorded at the welding axis and was associated with significant grain refinement, consistent with grain boundary strengthening described by the Hall–Petch relationship. In contrast, the highest hardness in the TIG joint (~110 HV2) was observed near the fusion boundary and was primarily associated with changes in the intermetallic particle system during the welding thermal cycle rather than with grain refinement.
4. RFW joints exhibited a substantially more favorable combination of mechanical properties than TIG-welded joints. The RFW joints achieved an ultimate tensile strength of approximately 247 MPa and an elongation to failure of 12.5%, whereas the corresponding values for the TIG joints were approximately 160 MPa and 1.7%, respectively. These results demonstrate that RFW preserves high ductility while maintaining excellent mechanical strength.
5. Fractographic analysis confirmed distinct differences in the fracture mechanisms of the two types of joints. The extruded material and the RFW joints exhibited a ductile fracture mechanism governed by the nucleation, growth, and coalescence of microvoids formed in the vicinity of intermetallic particles. In contrast, the TIG joints displayed a markedly reduced capacity for plastic deformation, which was associated with the formation of a cast microstructure and the microstructural changes occurring during melting and solidification.
6. Under the processing conditions investigated in the present study, Rotary Friction Welding (RFW) produced joints with a more favorable combination of microstructural characteristics, mechanical strength, and ductility than conventional TIG welding. These conclusions are valid for the selected welding parameters investigated in this work.

## Figures and Tables

**Table 1:** Chemical composition of the base material (AA2017) and filler metal (AlMg4.5Mn) used in this study (wt.%).

	Al	Si	Fe	Cu	Mn	Mg	Other
AA2017	93.45%	0.2%	0.2%	4.4%	0.9%	0.7%	≤0.15%
AlMg4.5Mn	93.16%	0.2%	0.4%	0.09%	1.0%	5.0%	≤0.15%

## Data Availability

The raw data supporting the conclusions of this article will be made available by the corresponding author on request.
